# “Without Filters” Nurse and Healthcare Worker Personal Protective Equipment Injuries and the COVID-19 Experience: An International Social Media Ethnographic Study

**DOI:** 10.3390/ijerph22111603

**Published:** 2025-10-22

**Authors:** Susan Solmos, Christine Eisenhauer, Robin Lally, Janet Cuddigan

**Affiliations:** 1 College of Nursing, University of Nebraska Medical Center, Omaha, NE 68198, USA; robin.lally@unmc.edu (R.L.);; 2 Ascension, St. Louis, MO 63134, USA; 3 Independent Researcher, Norfolk, NE 68701, USA

**Keywords:** personal protective equipment, PPE, COVID-19, PPE pressure ulcers/injuries, PPE injuries, nurses’ experiences, healthcare workers’ experiences, social media ethnography, qualitative study, pandemic preparedness, capacity building, respiratory protective devices, N95

## Abstract

Selfies of nurses and healthcare workers (HCWs) with painful personal protective equipment (PPE) injuries posted to social media provided early warning of the global PPE shortage impact during the COVID-19 pandemic. We aim to describe contextual factors associated with these injuries and describe factors that influenced posting on social media. A social media ethnographic study used purposeful sampling of Instagram posts (selfies/text) from March–October 2020 (170 posts; 26 countries). Posts were coded using focused content analysis to identify contextual factors. Data were reduced to understand and interpret the “essence” of the posts and discern themes. Themes included the following: (1) grueling shifts filled with unimaginable loss; (2) faces forever marked by the physical and emotional scars of COVID-19; (3) the COVID-19 battlefront; (4) dire and unprecedented PPE shortages; (5) pervasive fear (for self, colleagues, and family); (6) extreme emotional and physical consequences; (7) creating a collective voice. Examining injuries within the context of the nurses’/HCWs’ experiences provided new insights into the emotional scars, pervasive fears, and extreme emotional and physical consequences. An urgent need exists to address these harms and facilitate recovery. Before addressing emergency preparedness for the next pandemic event, psychosocial support is needed to address the harms incurred during the COVID-19 pandemic. Moreover, it is critical to understand past experiences to truly prepare for future pandemics.

## 1. Introduction

At the epicenter of the COVID-19 outbreak (Wuhan, China), shortages of personal protective equipment (PPE) led to over 1500 poorly protected nurses/HCWs contracting COVID-19 [1]. Multiple countries were soon overwhelmed with COVID-19 patients, exacerbating devastating shortages of PPE [2]. Even before the World Health Organization (WHO) designated COVID-19 as a pandemic, they warned that nurses/HCWs were endangered by rapidly depleting supplies [2]. Shortly thereafter, the devastating physical effects of extended-use PPE went viral on social media. A series of heart-rending images of nurses/HCWs with “bruised, exhausted selfies” posted to social media from Italy, Brazil, England, and the United States in mid-March 2020 garnered international media attention [3]. These alarming selfies detailing painful and potentially disfiguring facial injuries were a leading indicator of the consequences of dire global PPE shortages amidst unprecedented staffing demands.

A meta-analysis of 14 studies estimated the prevalence of adverse events associated with PPE use at 78%; reported physical adverse events included headaches, sleep disturbances, and dyspnea [4]. Adverse events were also associated with respirator-type mask and length of use [4]. In addition to physical symptoms, the emotional/psychological well-being of nurses/healthcare workers was impacted [5,6]. Common findings across studies included increased stress, anxiety, fear, isolation, and anger [5]. An umbrella review, including 44 meta-analyses, by Dragioti [6] found that mental health symptoms were common and had a profound negative impact on the mental health of HCWs during the pandemic. Almost one-third experienced anxiety/depression, approximately 40% insomnia/sleep problems, and 19% had post-traumatic stress disorder. Nurses had a higher prevalence of anxiety/depression and insomnia/sleep problems than doctors, while doctors had higher prevalence of stress and post-traumatic stress disorder [6].

Despite a plethora of studies examining the impact of the COVID-19 pandemic on nurses/HCWs, several notable gaps remain. Most studies were conducted in a single region or country with literature reviews providing the only global view of the pandemic. Heterogeneity of studies and low to moderate quality of studies remain limiting factors in meta-analyses [4,6]. Furthermore, recall limitation may have influenced the reporting of these experiences, as interviews or surveys largely occurred weeks or even months after the experiences due to the nature and severity of COVID-19 surges.

To our knowledge, no studies have examined the contemporaneous global experience of nurses/HCWs to determine factors which may influence PPE-related skin reactions/injuries. It is imperative to place these injuries within their historical, social, and cultural context [7] in order to understand the experience more fully from the unique point of view of frontline nurses/HCWs. How they perceived and made sense of these injuries and their experience caring for COVID-19 patients during the first few months of the pandemic is yet to be explored, highlighting the need to address this gap. Furthermore, although theorized [8,9], the unique personal, environmental, and community contextual factors associated with PPE-related skin reactions/injuries remain poorly understood, particularly on a global scale. Our research aimed to (1) describe the personal, environmental, and community contextual factors associated with PPE-related facial skin reactions/injuries and (2) describe what factors influence nurses/HCWs to share their experience of PPE-related facial injuries as articulated in Instagram posts. This is the first study to examine unprompted and unfiltered contemporaneous accounts posted by nurses/HCWs with PPE-related skin reactions/injuries during the first eight months of the pandemic.

## 2. Materials and Methods

### 2.1. Qualitative Approach

**Figure 1:**
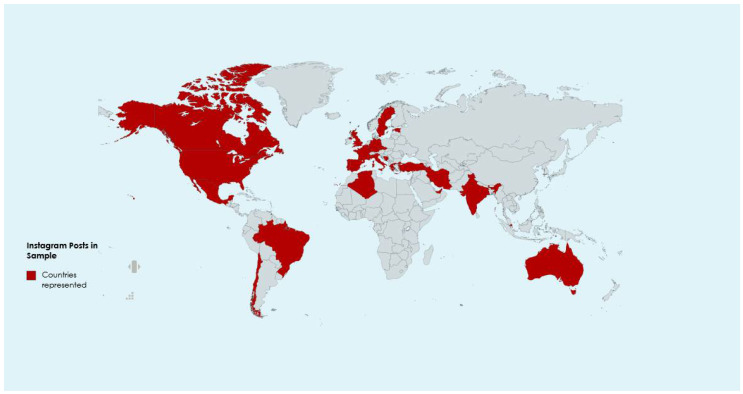
Countries represented in sample.

This social media ethnographic study used 170 Instagram posts (selfies/text) from 26 countries on six continents (see Figure 1). We examined the contextual setting with which the narratives were situated [10] to gain unique insights into the experiences by examining firsthand accounts on social media [11]. Instagram provided a platform for nurses/healthcare workers to sound the alarm and share experiences. In this aspect, social media/Instagram is uniquely suited to capturing the rich historical, temporal, and virtual account of their perceived experiences within the time and space that they unfolded [7,11]. Nurses/HCWs across the globe cared for COVID-19 patients under extreme conditions, sharing their stories in “socially constructed communities” [12]. Examining these PPE-related skin reactions/injuries within their unique historical, social, and cultural context [11] is imperative to fully appreciate and makes sense of these experiences, including how and why nurses/healthcare workers represented their experience on social media.

Researcher Characteristics. The principal investigator has an insider perspective [10], discovering this social community, #diariesfromthefield, while developing protocols for preventing PPE-related facial skin reactions/pressure injuries at an academic medical center in March 2020 [9]. Searching for images to guide the understanding and prevention of these PPE injuries led to an early #diariesfromthefield post.

Context. The meaning-making that nurses/HCWs assigned to their COVID-19 experiences occurred via stories sourced from an Instagram “socially constructed community” [12]. A constructivist paradigm guided our study, with the participants’ Instagram posts representing a “partial construction” of their social world [13]. Accordingly, the aims of this study were met using a novel virtual ethnographic approach [7,14,15], with a qualitative focused content analysis of the captions/text [16], to explore the socially constructed community and the created artifacts (social stories/posts). Our methodology incorporated emerging qualitative methods used to examine social media data [7]. Because qualitative methods applied to social media are relatively new and emerging, methods integrating traditional qualitative methods and social media analysis methods are explained in detail. Field notes were created throughout the observation of posts, data collection, and analysis to document the research team’s reflections, thoughts, and personal observations throughout the study [11].

### 2.2. Setting and Sample

Setting. The setting was a virtual and socially constructed community #diariesfromthefield on Instagram. Instagram, a “visual social media platform” [11] (p. 2) with over 1 billion active users across the globe [17], provided a rich source of data. Posts required both visual(s) and text.

Sampling strategy. Purposeful sampling of all posts, in any language, to the community #diariesfromthefield, with selfies displaying PPE-related skin reactions/injuries, occurred from March 2020 through October 2020. By October 2020, the confirmed deaths from COVID-19 surpassed 1 million globally [18] while PPE supply constraints were beginning to resolve, with supplies allocated based on need [19]. Sampling was restricted to this community in line with our ethnographic approach, to remain relevant to stressors of the first months of the pandemic, and to ensure a manageable data set for analysis. The principal investigator was immersed in the field (Instagram community) by logging into the platform at least twice per day. Instagram allows multiple images per post; therefore, each image/selfie included with the post were reviewed. Posts were saved under an account developed for this study. Posts including videos, patient identification, and those without visible PPE-related skin reactions/injuries were excluded.

Through 31 October 2020, a total of 1000 posts were identified. Eight hundred and thirty posts not meeting criteria were excluded, and 170 posts (17%) met the criteria of one or more selfie(s)/image(s) demonstrating PPE skin reactions/facial injuries. As the majority of posts were from nurses, hereafter nurses/HCWs will be referred to as nurses.

Purposeful sampling of posts increased the likelihood that extant social media posts represented nurses who had experience caring for COVID-19 patients, though no contact was made with participants. This purposeful sampling provided an additional source of verification for the sample. While this may have introduced a form of sampling bias by eliminating nurses wearing PPE (injuries are not visible), it was necessary to ensure the representativeness of our sample.

These data collection methods were also a means of thickening in this social media research. Three features, contextuality (examining data in context/original setting), temporality (slow data collection process, lengthy engagement and immersion with the field), and flexibility, including revising the sample as the study progressed [20], were used. This process is inherently constructivist as it layers contextual information, active engagement with the field, and understanding of the experiences and meaning of the participants posts [20]. This approach was consistent with our aim of understanding the sense-making experience of participants through social media ethnography and served to thicken the data.

### 2.3. Ethics Statement

As a social media ethnographic study, artifacts/posts were the source of data, not human subjects. Minimal risk to users exists, and any posts including identifying patient information were excluded. There is no direct benefit to Instagram users; however, there is potential indirect social benefit to nursing/public health and nursing/public health scholarship. The study protocol was reviewed by the University of Nebraska Medical Center Institutional Review Board (IRB) and determined to be exempt (0268-22-EX). Following completion of this study, we sought consent for exemplar selfies. When selecting exemplar images for each theme, only posts meeting our sampling criteria were used; however, exemplar images that best captured the essence of the theme may/may not include a selfie with facial injuries. Consent was obtained for use of all selfies/images used within this publication.

### 2.4. Data Collection Methods and Instruments

General principles from previously published social media studies were used to guide data collection [20,21]. Manual extraction of data was employed, as this entails immersion in the field and thickens the data [20,22]. Following methods outlined by Lalancette and Raynaud [21] as well as Latzko-Toth and colleagues [20], individual posts were saved within the Instagram social platform (in an account created for this study) and screenshots of the posts were saved and labeled with date, location, and username [21]. Posts not written in English were translated within Instagram and screen shots of both the original and translated posts were saved.

Data Analysis. Individual posts with selfies/images and text were compiled into one document and uploaded into MaxQDA2022 (VERBI Software, Berlin, Germany) [23]. Posts were organized chronologically starting with the first identified post dated March 9 2020 and labeled as previously described. Principles of ethnographic analysis were used to ground the research team in the data collection and analysis [22].

Posts were read in chronological order as a means of immersing researchers in the data prior to analysis [24] and in keeping with our ethnographic approach. Memoing next to the corresponding portion of the post captured thoughts or impressions [24]. In viewing the posts, the overall mood/tone of the selfie, the location (work/not work) of the selfie, and congruency with the tone and implicit or explicit purpose of the post were collected. Memoing as to what meaning the selfie was trying to convey in each post aligned with our research aims and was useful for insights at the next step of coding the data [24].

Qualitative Data Coding and Analysis. Using focused content analysis, we sought to gain greater descriptive clarity and contextual sensitivity into the factors identified in the Instagram posts (selfies/images and captions/texts) of nurses [16]. A codebook was created using initial a priori codes for contextual factors (personal, environmental, community) that we posited would influence the development of PPE-related skin reactions/facial injuries [8,25,26]. After research team discussion, emergent coding was used [27] to create codes not present in the codebook. Coding of the captions/texts occurred at the same time as coding of images. Double coding was used in instances when a segment of text could be linked to two codes [27]. During the course of coding, additional emergent codes were identified, resulting in subcategories or new codes [16,27]. Collapsing of codes and subcodes occurred following discussion by the principal investigator (initials redacted) and qualitative research expert (initials redacted) [27]. MaxQDA 2022 [23] computer analysis software was used to highlight and help code text. The holistic posts (selfie/image, text) were reduced by coding to examine the research purpose and aims, including the sense-making assigned by the nurse/healthcare worker posting.

Techniques to enhance trustworthiness. Images/selfies were analyzed alongside text and coded to determine if image(s) confirmed the interpretation/coding of the participant text. Images/selfies were coded for the presence of a healthcare setting or attire (e.g., scrubs) as another means of confirming the trustworthiness of the post. Photographs are generally viewed as a historical artifact, or establish that someone was a personal witness to an event. This was consistent with the ethnographic lens used for this study [28,29]. Selected posts (text and images) were reviewed weekly with a qualitative research (initials redacted) expert over a period of four weeks to discuss adequacy of coding, adherence to coding strategy, emergent codes, and other decisions.

Identifying themes. Data were reduced to understand and interpret the “essence” of the posts [24,27,30] and discern themes. Data reduction and theme development required consensus from the research team (redacted, redacted, and redacted) and alignment with research aims. Consensus was reached through discussion, review of stated research aims, and review of data.

## 3. Results

Nurses provided rich descriptions of their experiences during the COVID-19 pandemic. We analyzed 170 posts from 26 countries (Figure 1).

Ninety-eight percent of selfies/images portrayed nurses in their healthcare setting and/or wearing scrub attire. The majority (92.3%) of selfies/images had serious facial expressions with no smiles, and most often with their gaze directed at the viewer. Fatigue, evidenced by dark circles under eyes or physical appearance, was observed in 74% of posts. From usernames and/or content in posts, we confirmed nurses that were responsible for at least 64.7% of posts in the sample. We determined that selfies/images were congruent with the written posts and the time/space of the pandemic event as witnessed by nurses.

Our focused content analysis of community, personal, and environmental contextual factors resulted in seven themes: (1) grueling shifts filled with unimaginable loss; (2) faces forever marked by the physical and emotional scars of COVID-19; (3) the COVID-19 battlefront; (4) dire and unprecedented PPE shortages; (5) pervasive fear (for self, colleagues, and family); (6) extreme emotional and physical consequences; (7) creating a collective voice. In keeping with our social media ethnographic approach and as a means of immersing the reader in the experience and data thickening [31], entire quotes will be provided in Table S1 with exemplar quotes bolded.

### 3.1. Grueling Shifts Filled with Unimaginable Loss

**Figure 2:**
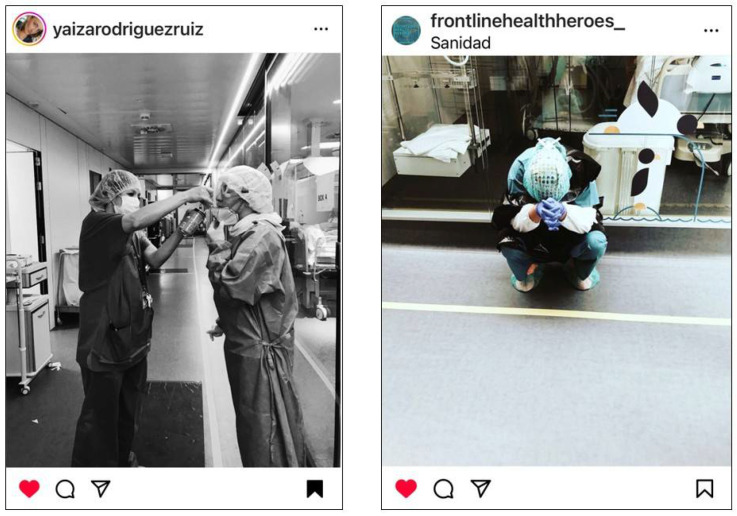
Grueling shifts filled with unimaginable loss. ©2020 @yaizarodriguezruiz. Used with permission. Left: “…*Between tears and sweat, my supervisor has to give me some Coca Cola…; I’ve been in there for 6 hours and I’m stuck in that outfit*…”.

Nurses described grueling shifts filled with unimaginable loss. They recounted the physical effects of long shifts coupled with unrelieved PPE use amidst the mounting death toll from COVID-19 (Figure 2).

*“… i’m honestly too tired, i have no energy left, these shifts are probably the worst we all had in our lives.*

*You People out there don’t realize how tragic and dangerous this situation is. …”*

—England

Nurses described the fatigue involved in caring for volumes of COVID-19 patients as well as the sheer number of deaths, both young and old, secondary to COVID-19.

*“…I work on a surgical unit, which was converted into one of the first COVID units in my hospital. I took my first COVID patient almost 2 months ago, and it feels like it’s been about 12 years since I’ve taken care of anything other than COVID. I have seen more death in 2 months than I have in my 2 short years as a nurse. After most of my shifts, I go home and cry. While many of the patients are elderly with comorbidities, putting them at higher risk of dying, it was not their time to go…”*

—USA

Nurses often recounted the physical effects of working long hours in PPE which provided additional context to both the high acuity and mortality of COVID-19 patients, as well as the severe strain on the healthcare system.

*“…I have never been so worn out from an overnight shift. Each time before I walk into a different patients room, I have to regown into PPE. I can’t count how many times I’ve done this last night. My hands are dried out. I am wearing a #N-95 mask I can barely breathe in and a second mask over it the whole shift. I am sweating wearing a gown, white body suit, shoe covers, switching gloves every 5 minutes, a hat, and goggles sticking to my face for 8 hours straight.*

*Last night, i heard “code blue-COVID” 2 times during my shift within 2 hours.*

*Now I’m scared. Our hospital has 2 floors flooded with 25 positive COVID patients each. We’re running low on supplies, packing out our ER and are running out of beds to admit patients because of this crisis. …”*

—USA

Grueling hours were often detailed, not only in terms of a single shift, but as excessive days of work as overwhelmed healthcare systems struggled to resource nurses to meet the surge of critically ill COVID-19 patients in “hot spots” around the globe.

*“…In march I’ve worked 27 days of 31!! I have to wrk this weekend… I hope I can do 4 days rest at the end of april…”*

—Italy

### 3.2. Faces Forever Marked by the Physical and Emotional Scars of COVID-19

**Figure 3:**
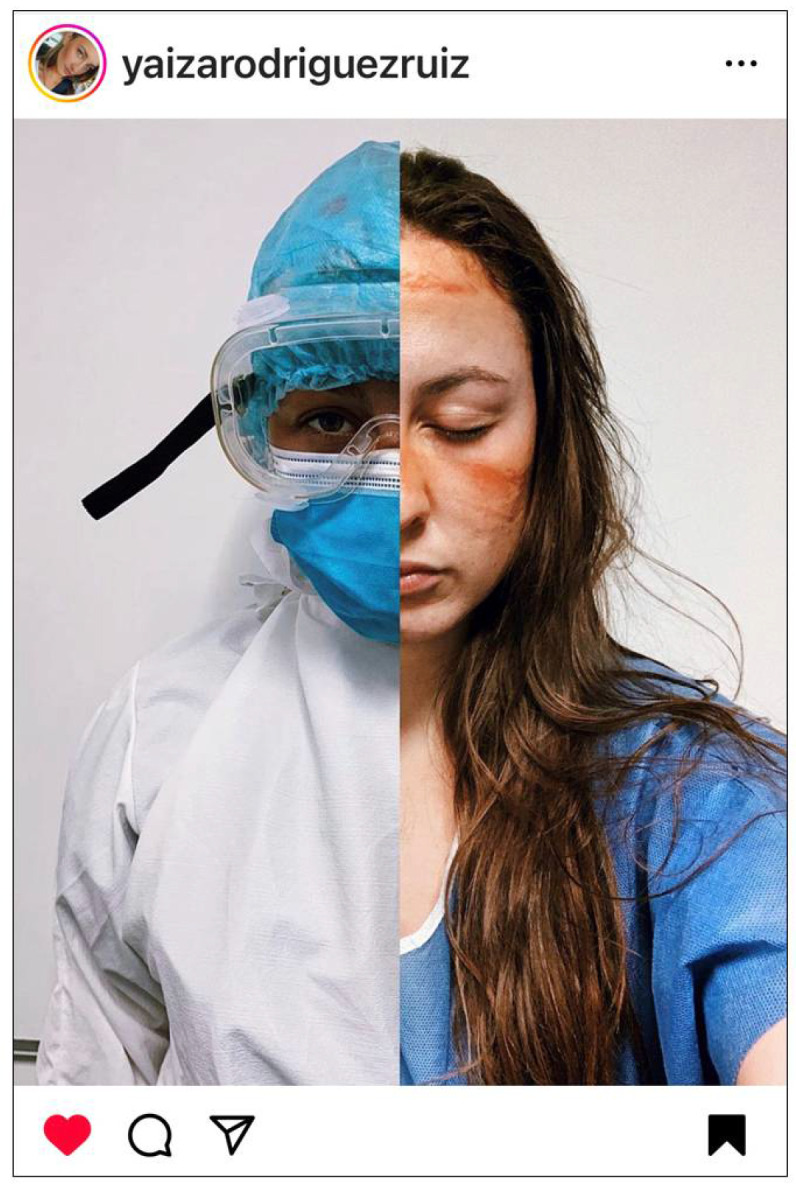
Faces forever marked by the physical and emotional scars of COVID-19. © 2020 @yaizarodriguezruiz. Used with permission.

This theme was described by many nurses. While selfies of PPE-related skin reactions/injuries provided a visible reminder of their physical injuries sustained during the care of COVID-19 patients, the emotional scars were central to their experience (Figure 3).

*“Back on day 2 when this nightmare began, all the health workers were struck by the injuries caused by the masks, glasses and other protections. Today day 36 of quarantine, those signs, wounds that have finally become scars that don’t matter, there are bigger scars in our hearts that no matter how much time passes, nothing, and no one can erase.…For every person we lose, a new scar opens. We will continue fighting so that those scars are smaller every day.”*

—Spain

One Italian nurse described crying secretly during her shift, as the “covids with severe breathing problems keep coming without respite.”

*“…we are giving it all and even more…we squeeze our teeth for physical and psychological pain, we cry secretly, But only 5 minutes, because then we have to be ready,…”*

—Italy

Nurses frequently used the term “*without filters*” to emphasize both that their selfies were unaltered but also in reference to the dire reality of their situation.

*“without filters. This is my face after 12 hours of night, masks that cut your nose with every breath, every yawn.*

*A face marked not only by physical pain but also by the pain of the mind, by the strength you must have to be next to these people who have seen their lives change, …*

*Patients who have sadness and awareness of the disease on their faces. …”*

—Italy

### 3.3. The COVID-19 Battlefront: The Fight for Supplies and the Truth

**Figure 4:**
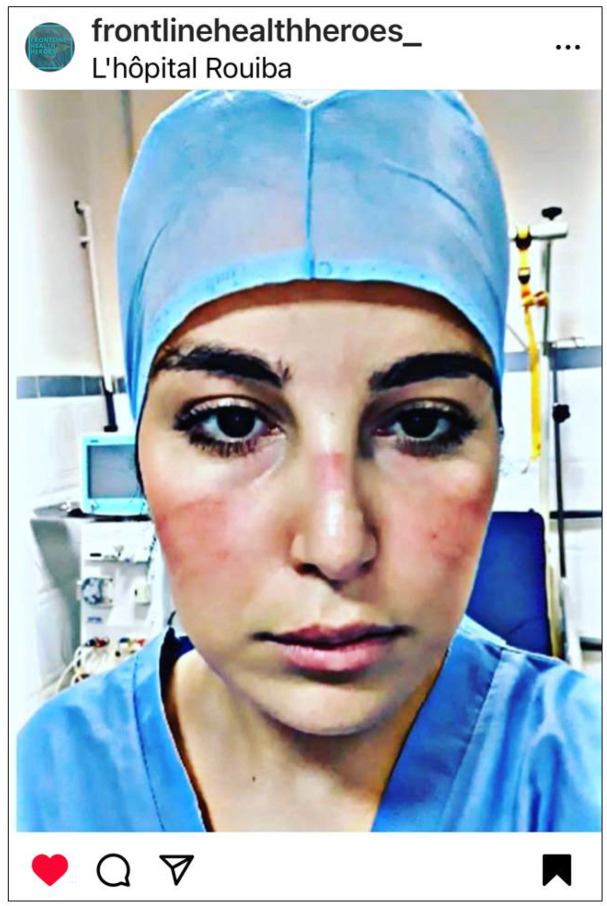
COVID-19 battlefront. © 2020 @younsi_ly. Used with permission. L′hôpital Rouïba; translation: Rouiba Hospital.

The community of Instagram users described their work as being on the frontlines of war, battling an unseen enemy (COVID-19). Social media was used to fight for essential supplies and to fight for truth amidst disinformation regarding the COVID-19 pandemic and social distancing measures (Figure 4). Direct requests for PPE donations to hospitals due to global shortages were common practice. Nurses also made direct requests to governments or politicians. PPE-related facial skin reactions/injuries were leveraged to demonstrate the effects of PPE shortages and the urgency of their requests for supplies.

*“This is what you look like after wearing an N95 mask all day. We don’t have enough of anything. We need @SenSanders…”*

—USA

As PPE shortages continued, nurses described purchasing industrial-use PPE from hardware stores as well as requesting donations from their communities.

*“…Being on the frontline working on a COVID19 unit in this national emergency, … We are taking this all on, doing the best we can with the supplies we’ve been given.*

*… Social media can be a blessing and a curse. The nurses and healthcare workers of this nation are trying to use it now to show just what we have been dealing with. We are not given the proper protective gear in order to protect ourselves while taking care of patients. Hospitals everywhere are running out of proper gear and supplies that are very much needed. Meaning healthcare employees are getting sick and contracting this deadly virus. The more employees that are out sick means the less employees we have to care for our patients. Many of my coworkers are out sick already and we haven’t even peaked yet. If you have access to getting #N95, respirators, surgical masks, face shields or ANY protective equipment please think about donating them to local hospitals to help protect us on the frontlines!*

*I urge you all to listen to what we have to say. We are seeing this all first hand…”*

—Canada

Fighting for citizens to respect social distancing guidelines and combat disinformation regarding COVID-19, nurses provided visible injuries while describing real world examples of the grueling shifts, overwhelmed healthcare systems, and the tragic circumstances in COVID-19 units while imploring people to stay home.

*“This is a picture of my sore and swollen face after wearing mask all day in ICU. You can hardly recognise me. I worked 39 hours in ICU this weekend. This is the sacrifice we specialist nurses have to give. …*

*People are dying lonely deaths. Their family cannot visit the ICU. ICU beds are full. We are overwhelmingly busy. Please stay home unless it’s absolutely essential. Maintain social distance of at least 2 meters apart. Avoid overcrowded lift or transportation. Coronavirus is real. Save the NHS.”*

—England

Although rich posts using the majority of the character count were common, short posts sounding the alarm were equally impactful. One nurse with severe PPE-related facial skin reactions/injuries simply stated “*#stayhome!!! There are no more beds.*”—Brazil

### 3.4. Dire and Unprecedented PPE Shortage

**Figure 5:**
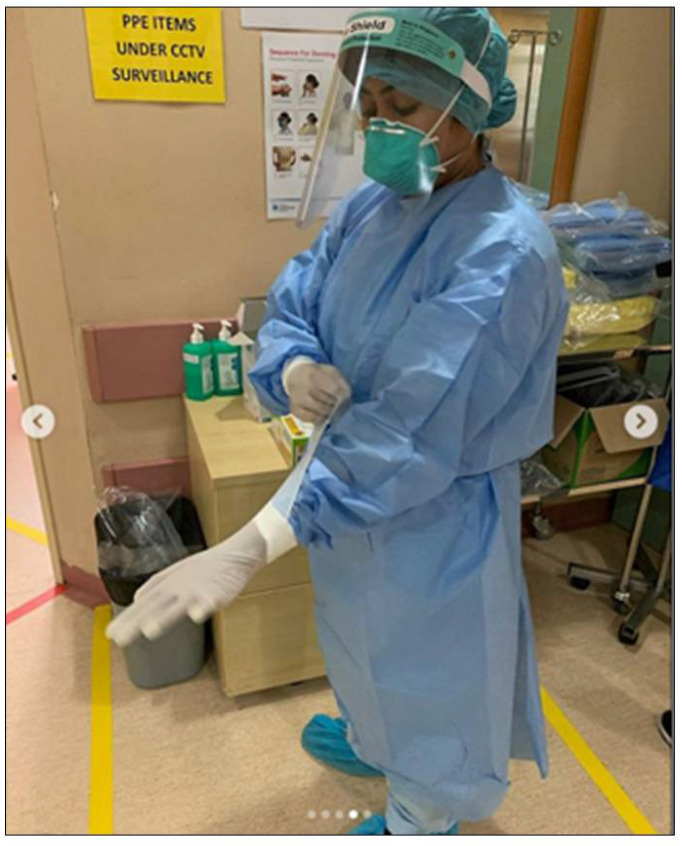
Dire and unprecedented PPE shortages. © 2020 @bobohuang0807. Used with permission.

During the COVID-19 pandemic unprecedented global PPE shortages required extended wear to conserve PPE, leading to painful PPE-related facial skin reactions/injuries and occupational COVID-19 exposure (Figure 5). As PPE shortages became dire, nurses described using the same respirator, not just for a single shift but for days of reuse. The risk of COVID-19 exposure due to PPE shortages was commonly acknowledged as a matter of fact.

*“…. Every single time we go to our patient’s room, we put on a hair cover, N95 mask, goggles, face shield, a protective gown, and shoe covers. When we come out of the room we take it off. We do this over and over again numerous times in a 12 hour shift. This is my face after 6 hours.*

*Supplies are running low. We are running out of N95 masks and are given 1 for our entire 12 hour shift. We place it in a plastic or paper bag and keep reusing it again and again. We can only hope that we are protecting ourselves enough. I’m not going to paint a pretty picture. It’s a struggle. …”*

—USA

One nurse noted that she had enough PPE to be “*safe(ish)*” while providing a stark description not only of extreme PPE shortages but of grueling working conditions.

*“Some numbers as my NYC assignment ends:*

*21 days, 12 hours a day, 20 patients a shift.*

*4 n95 masks.*

*Enough PPE to keep me safe(ish),…”*

—USA

Severe PPE shortages often left physicians/HCWs dangerously exposed to COVID-19 infection while performing high-risk procedures such as endotracheal intubation.

*“Dr. N [redacted for privacy] … collected donations of PPEs & gowns to give to his colleagues. Last week, this is N [redacted for privacy] about to intubate a patient, only wearing a patient’s gown because he couldn’t find PPE and the goggles leave these marks on his face.”*

—USA

Reuse of not only standard hospital PPE due to shortages, but the use of industrial or construction grade PPE was common.

*“In the covid ICU, I get 1 mask and it goes into a brown paper bag at the end of every shift, and gets used for the next shift.*

*Same hair cover and shoe covers for the whole shift. In and out of the covid + patient rooms at least 3 or 4 times an hour. *

*Wipe down the construction like face shield and use it again next shift. There’s talk of cleaning and reusing some PPE after it’s already been used in a covid + patient room and the environment…”*

—USA

### 3.5. Pervasive Fear of Contracting COVID-19; Putting Colleagues and Family at Risk

Days were marked by hypervigilance and awareness that common actions such as touching their faces or poor fitting PPE could have devastating consequences for themselves, colleagues, or families.

*“I am a nurse and I am facing this health emergency right now. I’m scared too, but not to go grocery shopping, I’m afraid to go to work. I’m scared because the mask might not fit my face well, or I may have accidentally touched myself with dirty gloves, or maybe the lenses don’t cover my eyes and something [sic COVID] might have passed. …”*

—Italy

**Figure 6:**
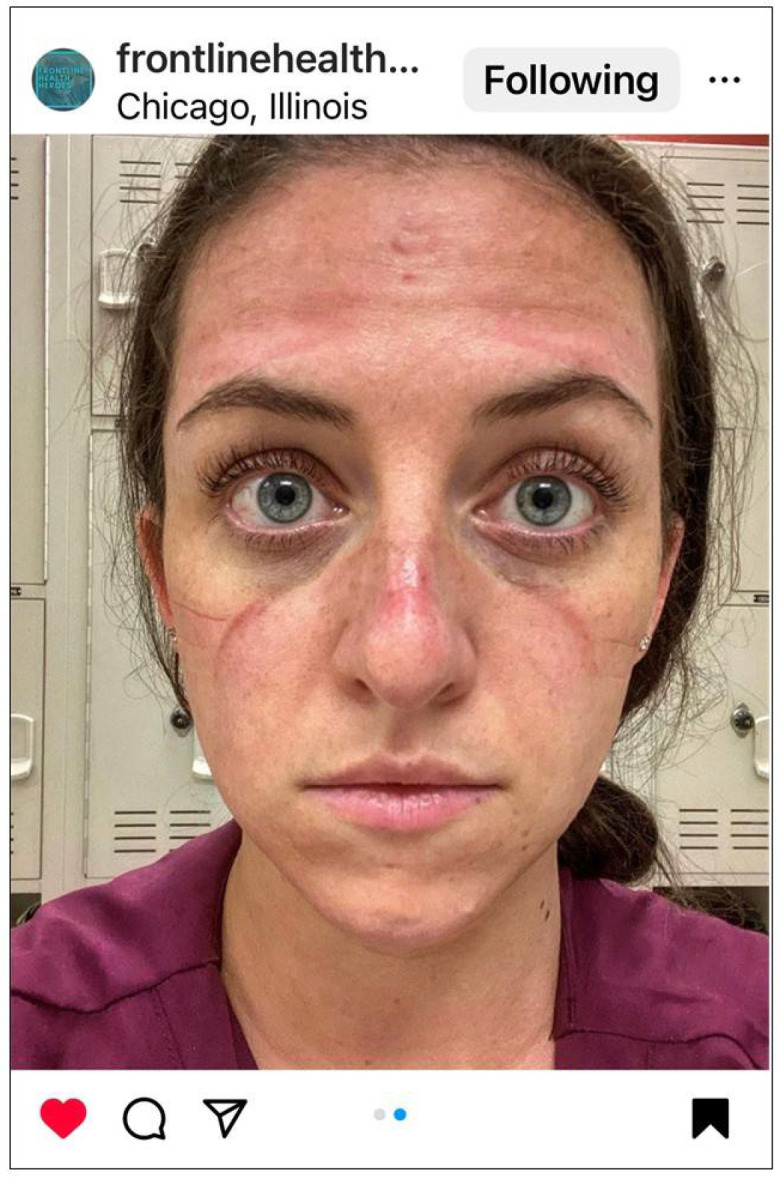
Pervasive fear of contracting COVID-19; putting colleagues and family at risk. © 2020 @allworth_fit Used with permission.

Descriptions of grueling hours coupled with intense physical discomfort from PPE without breaks or respite were coupled with hypervigilant awareness of the risk of contamination and COIVD-19 infection (Figure 6). As one ICU nurse described it,

*“hot, muggy …. feeling of shortness of breath, drops of sweat falling from the face, a face that you feel melt under the FP3 mask, the plastic glasses, the visor, the cap; wrapped in a waterproof gown… “The patient must be intubated” … “is desaturating” … “… you run, you continue to sweat … you prepare the drug with two pairs of gloves that limit your habitual hand movements … you sweat again and after hours you have no respite but you can’t drink, you can’t rest, you can’t pee dressed like that … In all this, the anxiety of being able to contaminate by making the gestures that you used to do before, this anxiety is the background to every maneuver, every thought, every action that you have to perform, you must constantly repeat to yourself that I could no longer touch your head if the elastic for your hair it hurts, if your nose itches you can bear, if you have that unbearable rebreathing in your mask you continue to breathe in it again and again and finish your work … ”*

—Italy

There was recognition of the risks of occupational COVID-19 exposure and serious illness or even death due to severe PPE shortages.

*“….An ER nurse in New York died today of COVID-19. He was in his 40s and had very mild asthma. That’s it. This is not just a tall tale, this is the real risk. I have to go into every patient’s room and in the back of my mind I think “this could be the patient that gets me sick… that kills me”.*

*“This could be the patient that gives me the virus I bring home to my children or asthmatic husband”.*

*This is my new reality.*

*But this is only the beginning. We haven’t even scratched the surface of the impact of what this illness is going to make on our country. And I’m scared.”*

—USA

For nurses working on designated COVID-19 units, fear was present throughout the shift, even during breaks, and extended to fear for their families.

*“…we’re scared to eat lunch in such a contaminated workspace, for fear of dying amongst the lifeless bodies surrounding us. How ironic that would be. And that’s all you think about. All shift. *

*When am I gonna get it.*

*My coworkers know my code status right?*

*Will I be the cause of my husbands death?*

*Kids? …”*

—USA

Fear was pervasive even after the shift ended. Exhausted after long shifts under grueling conditions, they took painstaking care to ensure that they were not exposing their families to COVID-19.

*“…When we finally get off work we can’t just come home and go to bed, we have to take every precaution to not spread this disease to our families,…”*

—USA

Countless others described isolating themselves from friends and family, staying in hotels, dormitories, or other living quarters to eliminate the risk of infecting their loved ones with COVID-19.

*“…. I have not been able to see the people I love for fear that I might inadvertently give them this disease. …”*

—USA

*“…#covid19 is different, so I’m much more scared for my family, scared to go to work, scared that I accidentally touch my face… afraid of bringing this virus home, afraid of contaminating my family! …”*

—Algeria

Anger and frustration at the untenable situation nurses faced daily were described, both in terms of working conditions and colleagues contracting COVID-19. Comments were directed at those not following social distancing guidelines.

*“While you’re bored and can’t stay at home, we are “falling” one by one as dominoes…”*

—Italy

### 3.6. Extreme Emotional and Physical Toll on Nurses and HCWs

**Figure 7:**
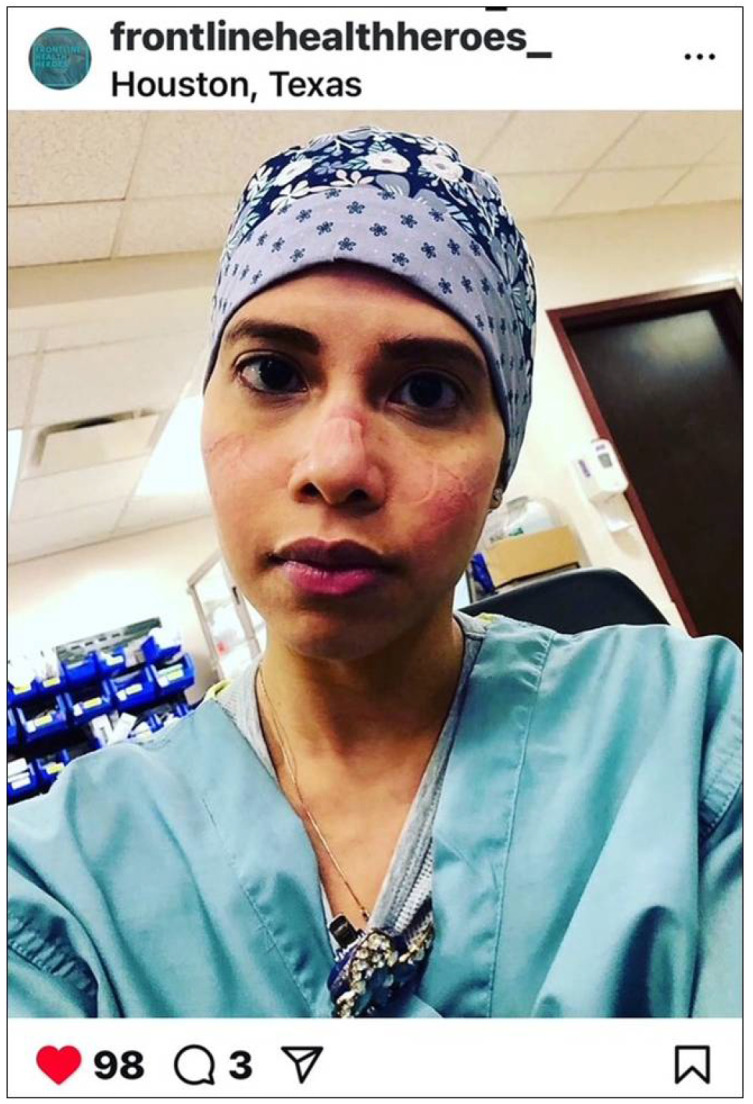
Extreme emotional and physical consequences. © 2020 @paulinemae31. Used with permission.

Nurses described their personal experiences, including the extreme emotional and physical toll of caring for patients during COVID-19 surges (Figure 7). Symptoms of anxiety, sleep disturbances, and being “physically and mentally drained” were commonly described.

*“… I don’t sleep at all, my hair falls out and it has turned gray, I lose weight, I have tachycardia when I go to sleep, I want to cry all the time, my hands and*

*Iface hurt, I have hurt myself on my back from scratching due to stress and I have muscle contractures and pain all over my body, as well as an exhaustion that sometimes leaves me in bed unable to move and some problems that I will have to solve when this is over. And I’m not one of those who is worse…”*

—Spain

The emotional and physical toll seemed to be inseparable and often exacerbated by isolation procedures that left patients dying without family at the bedside.

*“I’ve been trying to think of captions that truly explain how I feel in regards to everything going on right now. And every time I think about it it’s just producing so much fucking anxiety, sadness, disappointment, fear… but instead we are drowning… each day comes with less support & more responsibility. The safety & security we had yesterday is gone the next day. We have no legal support to back us up as we continue to lose our rights as a nurse, as a human. We can’t escape the misery.*

*We go 13 hours at a time sweating in places we’ve never sweat before from the gowns & stale OR scrubs… faces & ears aching from the suffocating masks. Then we go & care for people who are dying alone, because there are no visitors allowed. The emotional burden truly rips at the caring heart of every nurse …”*

—USA

During the early months of the COVID-19 pandemic, there was no certainty or known trajectory for when the pandemic would end or for alleviation of PPE shortages, which added to the stressors of working in COVID-19 “hotspots”. One COVID-19 ICU nurse, after describing the extreme physical and emotional symptoms experienced by HCWs working shifts in a COVID-19 “hotspot”, poignantly stated “*…It affects us a lot. I just want this to end.*”

*“… I don’t care how many hours I spend in a box [sic COVID ward], with my glasses and mask on, with the great PPE’s (irony) that they give us, being hot, overwhelmed, stressed because you get a ticket to the upac (one of the ICUs) and you know when you enter but never when you leave… this damn pandemic that is taking away our sleep, causing us anxiety, tachycardia, nerves, fear ….*

*And this is the result from 2:45 a.m. to 7:54 a.m., even second-skin dressings do nothing for me.”*

—Spain

Adding to the emotional and physical toll of working a shift on a COVID-19 unit was the need to repeat the same experience, without respite, in the following days (and often weeks).

*“… Do not think that it is a walk [sic in the park] for us to face a shift of work in these conditions, physically and psychologically you come out almost destroyed, yet every day we are there, ready to fight again, for everyone, for Italy, for us and for you …”*

—Italy

Recognition that their circumstances were not likely to improve, at least in the short-term, weighed heavily on nurses/healthcare workers, as voiced by a paramedic.

*“This is the face of someone who just spent 9 h in personal protective equipment moving critically ill Covid19 patients around London.*

*I feel broken—and we are only at the start. I am begging people, please please do social distancing and self isolation.”*

—England

### 3.7. Creating a Collective Voice, the Shared COVID-19 Experience of Frontline Nurses and HCWs

When sharing their experiences within the Instagram community, nurses/healthcare workers shifted between their experience in the singular and the shared experience.

*“All of us health workers are very disappointed to see … If you saw what I’ve seen …Do you still remember that we risk our lives every day, we are exhausted, injured, with sleep problems, states of anxiety and stress? I think many people no longer even know why they go out to applaud at 8:00 p.m.”*

—Spain

The collective voice was also used to share experiences and advocate for social distancing, imploring the public (and often politicians) to follow public health guidance.

*“Mine and my colleagues faces are sore and we are run ragged. Mostly we are desperately trying to save lives. Please please #StayHomeSaveLives”*

—England

*“…when it’s over, all the health personnel will go out into the street to claim and demand that public health is not to be played with and I hope that you will also go out with us and continue to applaud us, cheering us on. Because that’s when we’ll need you the most!”*

—Spain

**Figure 8:**
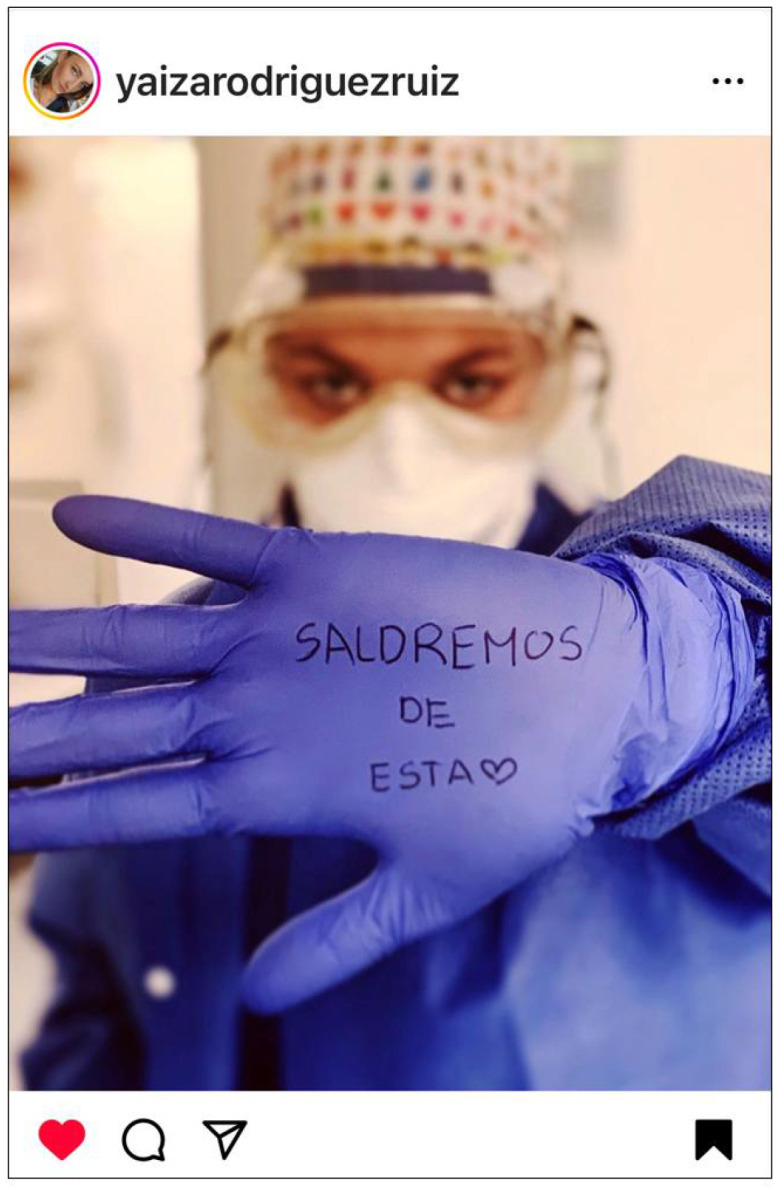
Collective voice. “WE WILL GET OUT OF THIS” © 2020 @yaizarodriguezruiz. Used with permission.

The collective voice described the shared challenges and expressed the professional pride and teamwork of nurses (Figure 8).

*“…I am psychologically tired, and as I am all my colleagues who have been in the same condition for weeks, but this won’t stop us from doing our job like we have always done. ….”*

—Italy

*“…so proud of my NHS team for stepping up to face the challenge …”*

—England

*“…we continue to go to work because we have to. Deep down we want to.*

*We want to help. It’s our calling. And I know that as nurses, nationally… globally… we will never stop doing what we are called to do. And we will die trying.”*

—USA

## 4. Discussion

Nurses across the globe shared their social story [15] of experiences during the first eight months of the COVID-19 pandemic in an Instagram community. This provided a poignant and powerful view of PPE-related facial skin reactions/injuries within the context of their experiences for this social media ethnographic study. Seven themes were identified: (1) grueling shifts filled with unimaginable loss; (2) faces forever marked by the physical and emotional scars of COVID-19; (3) the COVID-19 battlefront; (4) dire and unprecedented PPE shortages; (5) pervasive fear (for self, colleagues, and family); (6) extreme emotional and physical consequences; (7) creating a collective voice. These findings contribute to the existing knowledge of factors that contributed to PPE-related facial skin reactions/injuries [4,32] while underscoring that, although PPE-related facial skin reactions/injuries were highly visible physical consequences of PPE shortages, there are less visible emotional scars. The harm to nurses/healthcare workers cannot be fully addressed without understanding the hidden physical and emotional impact of their experience. While cross-sectional studies quantify the emotional and physical effects of working in COVID-19 hot spots, this innovative social media ethnographic study provides an unfiltered and first-hand account of the untenable situations nurses faced. Furthermore, our findings demonstrate that social media was used to “sound the alarm”, raising awareness of their plight and the essential role of public health and policies in saving the lives of patients and the nursing workforce alike.

As the World Health Organization (WHO) prepares for the next pandemic event [33], our study provides unique insights into how social media can be used to inform early identification of physical and emotional harm to nurses/healthcare workers and to inform global responses. Undoubtedly, the COVID-19 pandemic identified major gaps in emergency preparedness and the ability of nations and healthcare systems to respond to a pandemic event [34]. Vindrola-Padros and colleagues [35] described the monitoring of social media sites, which prompted a national response to improve nurses’ working conditions in a timely manner, thereby preventing further adverse occurrences associated with extended-wear PPE. These findings suggest that social media could be monitored in future public health crises and pandemics to provide timely updates and align resources (PPE, staff) to protect nurses/healthcare workers from physical adverse events across the globe long before peer-reviewed studies are published.

Our findings aligned with multiple reviews and syntheses detailing nurse/healthcare workers’ experiences during the pandemic, including long work hours [5,36,37,38], volume of patient deaths [36], the physical and emotional toll of the pandemic [5,6,36,37,38,39], PPE shortages [4,36,38,39], fear [5,6,36], and frustration with public response [5,36]. Risk factors associated with PPE-related facial skin reactions/injuries in the literature, including long work hours [4,5,36,38], extended PPE wear times [4], and fear [5,6,36], were identified as themes in our study. Although discomfort and pain associated with PPE use and PPE-related facial skin reactions/injuries were prevalent findings in the literature [4,36] and prominent in the selfies/images, surprisingly PPE-related skin reactions/injuries were seldom the posts’ focus; rather they served to provide context to the post.

In our study, grueling work hours were described in terms of both the intensity of a single shift as well as within the context of days or weeks of grueling shifts without respite. These findings are consistent with previously reported reviews reporting long intense work hours [4,5,36,38]. Within the context of selfies demonstrating PPE-related skin reactions/injuries, our findings align with a meta-analysis of studies from 16 countries which found that PPE-related skin reactions/injuries were associated with longer duration of PPE wear time, higher grade (more protective) PPE, and consecutive days of PPE use [4].

The impact of grueling hours amidst dire PPE shortages impacted the overall physical well-being of nurses/healthcare workers, as noted across three of our themes (grueling hours, physical scars, physical consequences). In contrast to quantitative findings, the collective voice of nurses across the globe provided powerful and contemporaneous insights into their experiences, highlighting both the physical and emotional toll. Investigators have quantified physical complaints, including discomfort/pain from PPE, headaches, shortness of breath and other physical symptoms. These physical complaints were often a result of or exacerbated by severe shortages of PPE, requiring use of industrial PPE, extended wear times, and quite often reuse of PPE [4,5,36,38].

The COVID-19 pandemic has profoundly impacted the emotional health of nurses/HCWs across the globe. As one nurse voiced, “…there are bigger scars in our hearts that no matter how much time passes, nothing, and no one can erase.” This is consistent with reviews that identified moral distress [38], negative impact on emotional well-being [5,6,36,39], and volume of deaths [36,40]. A pervasive fear for self, family, and colleagues was identified in our study, similar to findings from a meta-analysis of 173 studies reporting that 71% of nurses and HCWs experienced fear-related symptoms [6]. These findings underscore the need to provide mental health services during and after a public health crisis or pandemic. Monitoring social media can be used to identify early indicators of the need to provide emotional support to groups or individuals in real-time [35].

In terms of factors contributing to PPE-related skin reactions/injuries, our findings support that the fear of contagion may have led to over-tightening of PPE straps and reluctance to remove for breaks, thereby extending wear time [8,9] which was certainly exacerbated by PPE shortages and work conditions. Over-tightening aside, manufacturer’s one-size-fits-most approach to PPE design and shape combined with the relatively stiff materials used [41] is a factor in PPE injury development when coupled with the grueling hours of unrelieved wear-time described in our study. Investigators conducting an experimental evaluation of biomechanical factors (force, temperature, sub-epidermal moisture) attributed to device-related pressure injuries found significant increases in temperature and subepidermal moisture after healthy volunteers wore respirators for 2 h compared to baseline. Force levels under the respirator were notably higher with use of a respirator, while all measures were lower with use of a surgical mask [41]. There remains an urgent and unmet need for manufacturer’s to address PPE adverse events and redesign PPE to ensure the safety of those on the frontlines before the next pandemic.

Our findings demonstrate that nurses/HCWs used the power of social media to combat disinformation (COVID-19 battlefront), advocate for PPE and social distancing (COVID-19 battlefront, collective voice), and to share their experiences personally and collectively (collective voice). In contrast to findings from Vindrola-Padros and colleagues [35], who observed that social media was used to share guidelines amongst professionals, Instagram users in our study sought to inform the general public and document their experiences.

Limitations and Strengths. This study was limited to Instagram users and specifically those with PPE-related facial skin reactions/injuries and thus may not represent all nurses/HCWs. Posts were treated as extant artifacts; therefore, there was no opportunity to clarify intentions or ask additional questions. A major strength of this study was the use of global Instagram posts captured as the events occurred which limits recall bias [42] and provided access to a global sample experiencing the COVID-19 pandemic. The ability to translate posts to English within the Instagram app provided a unique opportunity to examine this global sample. The inclusion only of posts with PPE-related facial skin reactions/injuries enhanced the trustworthiness of the experiences. Moreover, the unfiltered and unprompted experiences provide unique insights that cross-sectional survey data fail to capture.

## 5. Conclusions

This study is innovative [43] in its use of social media posts as artifacts; using descriptive diary accounts of healthcare workers captured the environmental impact of the COVID-19 front lines in place and time. Our social media ethnographic study has the potential to advance science in public health and nursing by (1) using social media for data collection, (2) applying visual research methods to public health/nursing scholarship, (3) identifying a novel approach to reaching participants during an event as it unfolds (a global event in this instance), (4) using a small data approach, (5) providing a holistic view of PPE-related facial skin reactions/injuries including nurses’/HCWs’ experiences, and (6) ultimately providing insight as to what influenced nurses/HCWs to post on social media. Inarguably, the COVID-19 pandemic will have a far-reaching impact for the generations of nurses/HCWs that follow, and this study provides valuable insight into nurses’/HCWs’ physical, mental, and social environments amidst the early months of the COVID-19 pandemic. Our findings may influence future public health efforts, emergency preparedness response, needed emotional and physical support for nurses/HCWs during pandemics or other public health crises, impacts of PPE shortages on mental and physical well-being, effects of PPE design that failed to meet the needs for extended wear times, and an urgent unmet need for PPE redesign.

While this study has the power to inform future public health efforts, there remains a demonstrated need to provide ongoing support to nurses/HCWs whose traumatic experiences during the COVID-19 pandemic cannot be ignored. Nurses/HCWs have been recognized for their dedication, compassion, and expertise caring for patients during the pandemic. This recognition is well-deserved but falls short of addressing the needs of the nursing/HCW workforce to recover from their experiences [44,45]. Global nursing and physician shortages, present prior the pandemic, have been exacerbated as nurses/HCWs left the profession. Psychosocial support is urgently needed for the nursing/HCW workforce, not only as a retention strategy, but to ensure that nurses/HCWs are able to meet the ongoing demands of the profession and as preparedness for future pandemics. Despite WHO acknowledgement of the critical need to ensure nurses/HCWs have access to occupational mental health services [46], a mere 20% of 137 countries in a recent analysis had national laws or policies addressing occupational mental health service packages [47]. The physical injuries from PPE use may have faded, but the *emotional scars of COVID-19* likely remain and must be addressed. Understanding and addressing this trauma is critical to healing for many nurses/HCWs and for prevention of future trauma in the event of another pandemic. 

## Data Availability

The original data presented in the study are openly available on Instagram #diariesfromthefield.

## Supplementary Materials

The following supporting information can be downloaded at: https://www.mdpi.com/article/10.3390/ijerph22111603/s1, Table S1: Theme and Exemplar (in bold) with Entire Quote for Data Thickening.

## Abbreviations

The following abbreviations are used in this manuscript:

HCW	Health care worker
PPE	Personal protective equipment

## References

[1] Buckley B., Wee S., Qin A. China’s Doctors, Fighting the Coronavirus, Beg for Masks. *The New York Times*, 14 February 2020. https://www.nytimes.com/2020/02/14/world/asia/china-coronavirus-doctors.html.

[2] World Health Organization (WHO) (2020). Rational Use of Personal Protective Equipment (PPE) for Coronavirus Disease (COVID-19). https://apps.who.int/iris/bitstream/handle/10665/331498/WHO-2019-nCoV-IPCPPE_use-2020.2-eng.pdf?sequence=1&isAllowed=y.

[3] Law T. Health Care Workers Around the World Are Sharing Bruised, Exhausted Selfies After Hard days Treating COVID-19 Patients. *TIME*, 22 March 2020. https://time.com/5807918/health-care-workersselfies-coronavirus-covid-19/.

[4] Galanis P., Vraka I., Fragkou D., Bilali A., Kaitelidou D. (2021). Impact of personal protective equipment use on health care workers’ physical health during the COVID-19 pandemic: A systematic review and meta-analysis. Am. J. Infect. Control..

[5] Chemali S., Mari-Sáez A., El Bcheraoui C., Weishaar H. (2022). Health care workers’ experiences during the COVID-19 pandemic: A scoping review. Hum. Resour. Health.

[6] Dragioti E., Li H., Tsitsas G., Lee K.H., Choi J., Kim J., Choi Y.J., Tsamakis K., Estradé A., Agorastos A. (2022). A large-scale meta-analytic atlas of mental health problems prevalence during the COVID-19 early pandemic. J. Med. Virol..

[7] Sloan L., Quan-Haase A. (2016). The SAGE Handbook of Social Media Research Methods.

[8] Gefen A., Ousey K. (2020). Update to device-related pressure ulcers: SECURE prevention. COVID-19, face masks and skin damage. J. Wound Care.

[9] National Pressure Injury Advisory Panel (NPIAP) (2020). NPIAP Position Statements on Preventing Injury with N95 Masks. https://npiap.com/page/COVID-19Resources.

[10] Creswell J.W., Creswell J.D., Creswell J.W., Creswell J.D. (2018). Research Design: Qualitative, Quantitative, and Mixed Methods Approaches.

[11] Laestadius L. (2016). Instagram. The SAGE Handbook of Social Media Research Methods.

[12] Anderson B.R.O. (2006). Imagined Communities: Reflections on the Origin and Spread of Nationalism.

[13] Hand M. (2016). Visuality in social media: Researching images, circulations and practices. The SAGE Handbook of Social Media Research Methods.

[14] Hine C. (2017). Online social networks: Concepts for data collection and analysis. The SAGE Handbook of Online Research Methods.

[15] Murthy D. (2012). Digital ethnography: An examination of the use of new technologies for social research. SAGE Internet Research Methods.

[16] Hsieh H.F., Shannon S.E. (2005). Three Approaches to Qualitative Content Analysis. Qual. Health Res..

[17] Instagram About Us. https://about.instagram.com/about-us.

[18] World Health Organization (WHO) Weekly Operational Update on COVID-19-30 October 2020. https://www.who.int/publications/m/item/weekly-operational-update---30-october-2020.

[19] The Yellow House LLC (2021). Covid-19 Supply Chain System Assessment Comprehensive Analysis.

[20] Latzko-Toth G., Bonneau C., Milette M. (2017). Small data, thick data: Thickening strategies for trace-based social media research. The SAGE Handbook of Online Research Methods.

[21] Lalancette M., Raynauld V. (2019). The Power of Political Image: Justin Trudeau, Instagram, and Celebrity Politics. Am. Behav. Sci..

[22] Kozinets R., Dolbec P., Earkey A. (2014). Netnographic analysis: Understanding culture through social media data. The SAGE Handbook of Qualitative Data Analysis.

[23] (2020). MAXQDA2022.

[24] Creswell J.W., Poth C.N. (2018). Qualitative Inquiry and Research Design.

[25] EPUAP, NPIAP, PPPIA (2019). Prevention and Treatment of Pressure Ulcers/Injuries: Clinical Practice Guideline: The International Guideline.

[26] Jiang Q., Song S., Zhou J., Liu Y., Chen A., Bai Y., Wang J., Jiang Z., Zhang Y., Liu H. (2020). The Prevalence, Characteristics, and Prevention Status of Skin Injury Caused by Personal Protective Equipment Among Medical Staff in Fighting COVID-19: A Multicenter, Cross-Sectional Study. Adv. Wound Care.

[27] Saldaña J. (2021). The Coding Manual for Qualitative Researchers.

[28] Weilenmann A., Hillman T. (2020). Selfies in the wild: Studying selfie photography as a local practice. Mob. Media Commun..

[29] Chalfen R. (2020). Looking Two Ways: Mapping the Social Scientific Study of Visual Culture. The SAGE Handbook of Visual Research Methods.

[30] Roulston K. (2014). Analysing Interviews. The SAGE Handbook of Qualitative Data Analysis.

[31] Eldh A.C., Årestedt L., Berterö C. (2020). Quotations in Qualitative Studies: Reflections on Constituents, Custom, and Purpose. Int. J. Qual. Methods.

[32] Su H.H., Zhu F.F., Zeng H.L., Kong Y., Zhou H.J. (2023). Influencing factors of medical device-related pressure ulcers in medical personnel during the COVID-19 pandemic: A systematic review and meta-analysis. J. Tissue Viability.

[33] World Health Organization (WHO) WHO Pandemic Agreement. https://apps.who.int/gb/ebwha/pdf_files/WHA78/A78_R1-en.pdf.

[34] Ghebreyesus T.A. WHO Director-General’s opening remarks at media briefing—6 April 2023. Proceedings of the Media Briefing.

[35] Vindrola-Padros C., Andrews L., Dowrick A., Djellouli N., Fillmore H., Gonzalez E.B., Javadi D., Lewis-Jackson S., Manby L., Mitchinson L. (2020). Perceptions and experiences of healthcare workers during the COVID-19 pandemic in the UK. BMJ Open.

[36] Fernández-Basanta S., Espremáns-Cidón C., Movilla-Fernández M.J. (2022). Novice nurses’ transition to the clinical setting in the COVID-19 pandemic: A phenomenological hermeneutic study. Collegian.

[37] Galanis P., Vraka I., Fragkou D., Bilali A., Kaitelidou D. (2021). Nurses’ burnout and associated risk factors during the COVID-19 pandemic: A systematic review and meta-analysis. J. Adv. Nurs..

[38] Zulaihah S., Harmayetty H., Kusumaningrum T. (2022). Factors Related to Nurses’ Moral Distress in the Era of the COVID-19 Pandemic: A Literature Review. Crit. Med. Surg. Nurs. J..

[39] Turner S., Botero-Tovar N., Herrera M.A., Kuhlmann J.P.B., Ortiz F., Ramírez J.C., Maldonado L.F. (2021). Systematic review of experiences and perceptions of key actors and organisations at multiple levels within health systems internationally in responding to COVID-19. Implement. Sci..

[40] Nikbakht Nasrabadi A., Abbasi S., Mardani A., Maleki M., Vlaisavljevic Z. (2022). Experiences of intensive care unit nurses working with COVID-19 patients: A systematic review and meta-synthesis of qualitative studies. Front. Public Health.

[41] Peko L., Ovadia-Blechman Z., Hoffer O., Gefen A. (2021). Physiological measurements of facial skin response under personal protective equipment. J. Mech. Behav. Biomed. Mater..

[42] Kjellsson G., Clarke P., Gerdtham U.G. (2014). Forgetting to remember or remembering to forget: A study of the recall period length in health care survey questions. J. Health Econ..

[43] Kuruvilla S., Mays N., Pleasant A., Walt G. (2006). Describing the impact of health research: A Research Impact Framework. BMC Health Serv. Res..

[44] Catton H., Iro E. (2021). How to reposition the nursing profession for a post-covid age. BMJ.

[45] Boniol M., Kunjumen T., Nair T.S., Siyam A., Campbell J., Diallo K. (2022). The global health workforce stock and distribution in 2020 and 2030: A threat to equity and ‘universal’ health coverage?. BMJ Glob. Health.

[46] World Health Organization (WHO) Accelerating Action on the Global Health and Care Workforce by 2030. 2025, 6. https://apps.who.int/gb/ebwha/pdf_files/EB156/B156_CONF14-en.pdf.

[47] Kavanagh M.M., Radhakrishnan A., Unnikrishnan V., Cometto G., Kane C., Friedman E.A., Srivatsan V., Gil Abinader L., Campbell J., Health & Care Worker Policy Lab (2025). Correction: Laws for health and care worker protection and rights: A study of 182 countries. PLOS Glob. Public Health.

