# Supplementary material for: “Without Filters” Nurse and Healthcare Worker Personal Protective Equipment Injuries and the COVID-19 Experience: An International Social Media Ethnographic Study
Source: Int J Environ Res Public Health. 2025 Oct 22;22(11):1603. doi: 10.3390/ijerph22111603 (PMC12652312; doi:10.3390/ijerph22111603)
Supplement: Supplementary file 1 [file ijerph-22-01603-s001.zip › ijerph-3906837-supplementary.pdf]

Table S1. Theme and Exemplar (in bold) with Entire Quote for Data Thickening

**Theme**	**Entire Quote for Data Thickening; Exemplar From Text in Bold (Month/Year and Country)**
**Grueling shifts filled with unimaginable loss**	April 2020, England<br><br>"We have always held to the hope, the belief, the conviction that there is a better life, a better world, beyond the horizon.<br>Franklin D. Roosevelt" ✨✨✨<br><br>*"I wanted to try to explain what feels like being an ITU nurse in this moment.*<br><br>*But* ***i'm honestly too tired, i have no energy left, these shifts are probably the worst we all had in our lives.***<br><br>***You People out there don't realize how tragic and dangerous this situation is.***<br>*so PLEASE be careful.*<br><br>*And help us taking care of you! -a tired and ugly intensive care nurse* 💉🩺*"*
	April 2020, USA<br><br>"I think by now you all know I'm a nurse, and most of you are probably sick of seeing me in scrubs with my new busted face. Which is fine, I get sick of myself too sometimes. As a nurse, it's my job to advocate for my patients, so that's what I'm going to do. I've been seeing so much bullshit lately, so many angry Americans protesting and spewing their idiotic agendas. Freedom of speech is a great thing, but uneducated takes on what is actually happening in the world is not. I fully admit I was apprehensive about this virus- everything I read made me think it was just a hyped up flu. **I work on a surgical unit, which was converted into one of the first COVID units in my hospital. I took my first COVID patient almost 2 months ago, and it feels like it's been about 12 years since I've taken care of anything other than COVID. I have seen more death in 2 months than I have in my 2 short years as a nurse. After most of my shifts, I go home and cry. While many of the patients are elderly with comorbidities, putting them at higher risk of dying, it was not their time to go.** I am sick & tired of these protestors, these conspiracy riddled morons, walking around with their signs downplaying the tragedy we've all

	been living, just because they're bored in their fucking houses. You are taking away so much from the people who have lost loved ones they couldn't say goodbye to. So I'm speaking for them- for my patients who have died, for the ones who have recovered and gone home, and for the ones we're still trying to help get back on their feet. Please stay home, please let us continue to heal. The longer you do so, the sooner we can get back to normal ❤️"
	March 2020, USA<br><br>"... #Our hospital is being turned upside down to prepare for #COVID-19 patients. I live in New York and moved here for nursing school from #Massachusetts. New York is the top state with cases increasing each day and deaths occurring. I didn't take this #outbreak as serious before, I thought it was just like the flu. I have now realized how serious this is and how careful we have to all be to keep each other safe. I have my family back Massachusetts worried sick, calling me everyday and my dad calling me every hour to make sure I am okay. I have a 2 year old niece and it breaks my heart that I cannot see her because of the possibility of having it god forbid and not knowing.<br>**I have never been so worn out from an overnight shift. Each time before I walk into a different patients room, I have to regown into PPE. I can't count how many times I've done this last night. My hands are dried out. I am wearing a #N-95 mask I can barely breathe in and a second mask over it the whole shift. I am sweating wearing a gown, white body suite, shoe covers, switching gloves every 5 minutes, a hat, and goggles sticking to my face for 8 hours straight.**<br>**Last night, i heard "code blue-COVID" 2 times during my shift within 2 hours.** Now I'm scared. **Our hospital has 2 floors flooded with 25 positive COVID patients each. We're running low on supplies, packing out our ER and are running out of beds to admit patients because of this crisis.** I am risking my health and life by being face-to-face with patients and helping them as much as I can. I have to make sure I change my scrubs, wipe my shoes, throw them in a bag and run right to the shower when I get home. I love my boyfriend, he has a grandmother at home. I get nervous each time i walk into the house because I feel like I'm putting her and his family at risk.<br><br>It's our passion to help our patients live the longest, healthiest lives possible while receiving care. All I ask is please do your part by staying home and doing some serious social distancing."

	<u>March 2020, Italy</u><br><br>“Happy Easter… things seems [sic to be] going better but we’re tired. **In march I’ve worked 27 days of 31!! I have to wrk this weekend.. I hope ican do 4 days rest at the end of april** [sic praying hands emoji] I need it so much.. Thank you for your attention, for your post and your support…”
**Faces forever marked by the physical and emotional scars of COVID-19**	*<u>April 2020, Spain</u>*<br><br>***“Back on day 2 when this nightmare began, all the health workers were struck by the injuries caused by the masks, glasses and other protections. Today day 36 of quarantine, those signs, wounds that have finally become scars that don't matter, there are bigger scars in our hearts that no matter how much time passes, nothing, and no one can erase.*** *Every day we see family goodbyes without knowing if they will see each other again, we say goodbye to patients who had many plans for the future and a family waiting outside, we hold hands tightly, this being the best of treatments.* ***For every person we lose, a new scar opens. We will continue fighting so that those scars are smaller every day.”***
	<u>April 2020, Italy</u><br><br>While you’re bored and can’t stay at home, we are “falling” one by one as dominoes. The **covids with severe breathing problems keep coming without respite** and unfortunately now they are no longer just elderly, but many young people **we are giving it all and even more…we squeeze our teeth for physical and psychological pain, we cry secretly, But only 5 minutes, because then we have to be ready,** on the mark… for you!!!! But if you don’t make yours, we won’t make it, we can’t go on like this for a long time… It will come That day that, who will get sick, will no longer have healthcare staff in the right amout to be saved.. You or your loved ones. WHILE YOU GO OUT FOR A WALK OR A RUN OR DRINK A COFFEE “SO MUCH I’M CAREFUL.. SO MUCH NO ONE IS AROUND” THINK ABOUT THE FACT, THAT IF THIS STYLE IS NOT RECIDE, IT’S JUST FOR YOU Everyone deals with their own conscience. THINK ABOUT THIS [sic praying hands emoji]

	<u>April 2020, Italy</u><br><br>**“without filters. This is my face after 12 hours of night, masks that cut your nose with every breath, every yawn.**<br>**A face marked not only by physical pain but also by the pain of the mind, by the strength you must have to be next to these people who have seen their lives change,** even if momentarily.<br>**Patients who have sadness and awareness of the disease on their faces.**<br>Patients who, despite everything, still care about their family members outside.<br>This experience, even if I have recently been living it, has already changed my perception of things and priorities in life.<br>You realize how beautiful the wind that comes from a window can be while you look out.<br>You realize how nice it can be to wash your hands with fresh water.<br>You realize how many things you have taken for granted.”
**COVID-19 battlefront: the fight for supplies and the truth**	*<u>March 2020, USA</u>*<br><br>***“This is what you look like after wearing an N95 mask all day. We don’t have enough of anything. We need @SenSanders*** *more than ever now. Help us help you.”*
	<u>April 2020, Canada</u><br><br>“There is nothing in this world I see myself doing other than being a nurse. I knew from a young age running towards emergencies wasn’t for everybody.<br>**Being on the frontline working on a COVID19 unit in this national emergency**, I can say first hand nurses are not running away. **We are taking this all on, doing the best we can with the supplies we’ve been given.**<br>In times like this, **Social media can be a blessing and a curse. The nurses and healthcare workers of this nation are trying to use it now to show just what we have been dealing with**. **We are not given the proper protective gear in order to protect ourselves while taking care of patients. Hospitals everywhere are running out of proper gear and supplies that are very much needed. Meaning healthcare employees are getting sick and contracting this deadly virus. The more employees that are out sick means the less employees we have to care for our patients. Many of my coworkers are out sick already and we haven’t even peaked yet. If you have access to getting #N95, respirators, surgical masks, face shields or ANY protective equipment please think about donating them to local hospitals to help protect us on the frontlines!**

	**I urge you all to listen to what we have to say. We are seeing this all first hand.** Please stay home! If I was given the chance to stay home to protect my friends and family, I would. The quicker we stop this thing, the quicker we can get back to our "normal" life.<br>In these pictures, these ONE time use face mask/shield have been used over and over and over again. Meaning, we are probably being exposed to this virus with no other choice. Keep advocating for us! Stay safe out there everyone❤️ Thank you for reading, I love you all. And to all my kick ass coworkers, I appreciate you all. We will get through this together!"
	April 2020, UK<br><br>"My people thanks for all your compliments. You are all my strength. Please keep me in your prayers. **This is a picture of my sore and swollen face after wearing mask all day in ICU**. **You can hardly recognise me. I worked 39 hours in ICU this weekend. This is the sacrifice we specialist nurses have to give.** I am a trained anaesthetic and recovery nurse.<br><br>I am having a bit of rest and going back to care for my patients. **People are dying lonely deaths. Their family cannot visit the ICU. ICU beds are full. We are overwhelmingly busy. Please stay home unless it's absolutely essential. Maintain social distance of at least 2 meters apart. Avoid overcrowded lift or transportation. Coronavirus is real. Save the NHS**."
	April 2020, Brazil<br><br>#ficaemcasa **#stayhome!!! "There are no more beds"**
**Dire and unprecedented PPE shortages**	April 2020, USA<br><br>"This is what it means to be on the frontline. **Every single time we go to our patient's room, we put on a hair cover, N95 mask, goggles, face shield, a protective gown, and shoe covers. When we come out of the room we take it off. We do this over and over again numerous times in a 12 hour shift. This is my face after 6 hours.**<br>**.**<br>**Supplies are running low. We are running out of N95 masks and are given 1 for our entire 12 hour shift. We place it in a plastic or paper bag and keep reusing it again and again. We can only hope that we are protecting ourselves enough. I'm not going to paint a pretty picture. It's a struggle.**<br>.

	Two days ago I held my patient’s hand as she mouthed to me that she was scared (she is on a ventilator so she can’t talk). I kept telling her she’s going to get through this and to stay strong. She is young. She is alone. No family around because visitors are not allowed. Tears fill my eyes because the only thing I could do was keep reassuring her. This is the reality. If you think because you’re young and healthy that you won’t be affected. Think again. Not only are people dying, they are dying alone.<br>.<br>Please take this seriously. Please listen to all the healthcare workers who are seeing this firsthand. Please stay home. And If you are struggling right now I know the feeling. We are scared. We are sad. We are hopeful.”
	<u>April 2020, USA</u><br><br>**“Some numbers as my NYC assignment ends:**<br><br>**21 days, 12 hours a day, 20 patients a shift.**<br><br>**4 n95 masks.**<br><br>Unknown number of Covid positive patients. The facility I worked at hasn’t tested a patient in two weeks. I can tell you the number is high.<br>**Enough PPE to keep me safe(ish)**, but nowhere near enough to protect my patients.<br><br>And just over a day until I’m back in Texas.<br>Y’all, we really need to be careful.”
	<u>March 2020, USA</u><br><br>***“Dr. N [redacted for privacy]*** *- knew him since his intern year.* ***He collected donations of PPEs & gowns to give to his colleagues. Last week, this is N [redacted for privacy] about to intubate a patient, only wearing a patient’s gown because he couldn’t find PPE and the goggles leave these marks on his face.”*** *– USA*
	<u>April 2020, USA</u><br><br>**“In the covid ICU, I get 1 mask and it goes into a brown paper bag at the end of every shift, and gets used for the next shift.**

	• **Same hair cover and shoe covers for the whole shift. In and out of the covid + patient rooms at least 3 or 4 times an hour.** • **Wipe down the construction like face shield and use it again next shift.** • **There's talk of cleaning and reusing some PPE after it's already been used in a covid + patient room and the environment**. • We have been finding that a lot of patients are still testing positive and assumed to be contagious after the "14 days." • I'm emotional. I'm irritated. I'm annoyed. I'm worried. And I'm exhausted. But through all of that, it's really been an honor to take care of this patient population when they're at their most vulnerable. While each shift has its challenges, I'm grateful for the opportunity that each work day brings."
**Pervasive fear (for self, colleagues, and family)**	<u>March 2020, Italy</u><br><br>**"I am a nurse and I facing this health emergency right now. I'm scared too, but not to go grocery shopping, I'm afraid to go to work. I'm scared because the mask might not fit my face well, or I may have accidentally touched myself with dirty gloves, or maybe the lenses don't cover my eyes and something [sic COVID] might have passed.** I'm physically tired because protective devices hurt, scrubs are sweating and once dressed I can't go to the bathroom or drink for six hours. I am psychologically tired, and as I am all my colleagues who have been in the same condition for weeks, but this won't stop us from doing our job like we have always done. I will continue to care and take care of my patients, because I am proud and in love with my job. What I ask anyone reading this post is not to frustrate the effort we are making, to be selfless, to stay in the house and so protect those who are most fragile. We young people are not immune to coronavirus, we can get sick too, or worse we can make you sick. I can't afford the luxury of going back to my house in quarantine, I have to go to work and do my part. You all do [sic make] yours, I ask you please."

	<u>March 2020, Italy</u><br>***“hot, muggy .... feeling of shortness of breath, drops of sweat falling from the face, a face that you feel melt under the FP3 mask, the plastic glasses, the visor, the cap; wrapped in a waterproof gown... "The patient must be intubated" ... "is desaturating "...."*** *and hypothesis* ***"... you run, you continue to sweat ... you prepare the drug with two pairs of gloves that limit your habitual hand movements ... you sweat again and after hours you have no respite but you can't drink, you can't rest, you can't pee dressed like that .... In all this, the anxiety of being able to contaminate by making the gestures that you used to do before, this anxiety is the background to every*** **maneuver,** ***every thought, every action that you have to perform, you must constantly repeat to yourself that I could no longer touch your head if the elastic for your hair it hurts, if your nose itches you can bear, if you have that unbearable rebreathing in your mask you continue to breathe in it again and again and finish your work ..."***
	<u>March 2020, USA</u><br><br>“I broke down and cried today.<br>I cried of exhaustion, of defeat.<br>Because after 4 years of being an ER nurse, I suddenly feel like I know nothing.<br>Because my face hurts after wearing an N95 for 13 fucking hours, which happens to be the same N95 I wore yesterday for 12.5 hours, and the same one from all last week.<br><br>I don’t know how many times I’ve heard the statement “but this is what you signed up for”. Just, no.<br><br>I signed up to take care of sick patients, yes. I did not sign up to be unprotected by their sickness (although my hospital is busting their asses to try to protect us). I did not sign up to be yelled at by angry patients because our government failed to be prepared. I did not sign up to risk mine and my family’s health and safety because people wanted to go on their vacations after they said NOT to.<br>**An ER nurse in New York died today of COVID-19. He was in his 40s and had very mild asthma. That’s it. This is not just a tall tale, this is the real risk. I have to go into every patient’s room and in the back of my mind I think “this could be the patient that gets me**

	**sick... that kills me". "This could be the patient that gives me the virus I bring home to my children or asthmatic husband". This is my new reality.**<br>**But this is only the beginning. We haven't even scratched the surface of the impact of what this illness is going to make on our country.**<br>**And I'm scared."**
	<u>April 2020, USA</u><br><br>"I've been trying to think of captions that truly explain how I feel in regards to everything going on right now. And every time I think about it it's just producing so much fucking anxiety, sadness, disappointment, fear... this pandemic had such opportunity to empower nurses, collaborate, heal.. but instead we are drowning... each day comes with less support & more responsibility. The safety & security we had yesterday is gone the next day. We have no legal support to back us up as we continue to lose our rights as a nurse, as a human. We can't escape the misery.<br>We go 13 hours at a time sweating in places we've never sweat before from the gowns & stale OR scrubs... faces & ears aching from the suffocating masks. Then we go & care for people who are dying alone, because there are no visitors allowed. The emotional burden truly rips at the caring heart of every nurse.<br>And if we're lucky enough to get a break from it all, **we're scared to eat lunch in such a contaminated workspace, for fear of dying amongst the lifeless bodies surrounding us. How ironic that would be. And that's all you think about. All shift.**<br>**When am I gonna get it.**<br>**My coworkers know my code status right?**<br>**Will I be the cause of my husbands death?**<br>**Kids?**<br>We haven't even hit our peak yet but we're told it's coming. How envious we are of those "bored" people that are forced to have to work from home. But we continue to go to work because we have to. Deep down we want to.<br>We want to help. It's our calling. And I know that as nurses, nationally.. globally... we will never stop doing what we are called to do. And we will die trying."

	April 2020, USA<br><br>“THIS IS NOT EASY<br><br>THIS IS NOT A JOKE<br>A CUTE CATCH PHRASE<br>OR AN EXCUSE AS TO WHY YOU JUST NEED TO HANG OUT WITH THAT PERSON<br><br>THIS IS REAL<br>Night shifts in the ICU are hard. As nurses we invest all we have into our patients. We don’t take breaks, have time to eat or even time to sit because your health comes first.<br><br>**When we finally get off work we can’t just come home and go to bed, we have to take every precaution to not spread this disease to our families.**<br><br>WE ARE TIRED and we need your help!<br>Please stay home, it’s the least you can do ❤️”
	April 2020, Italy<br><br>**“While you’re bored and can’t stay at home, we are “falling” one by one as dominoes.** The covids with severe breathing problems keep coming without respite and unfortunately now they are no longer just elderly, but many young people we are giving it all and even more…we squeeze our teeth for physical and psychological pain, we cry secretly, But only 5 minutes, because then we have to be ready, on the piece… for you!!!! But if you don’t make yours, we won’t make it, we can’t go on like this for a long time… It will come That day that, who will get sick, will no longer have healthcare staff in the right amout to be saved.. You or your loved ones. WHILE YOU GO OUT FOR A WALK OR A RUN OR DRINK A COFFEE “SO MUCH I’M CAREFUL.. SO MUCH NO ONE IS AROUND” THINK ABOUT THE FACT, THAT IF THIS STYLE IS NOT RECIDE, IT’S JUST FOR YOU Everyone deals with their own conscience. THINK ABOUT THIS [sic praying hands emoji]”

	May 2020, USA<br><br>"This is me after wearing my PPE for only an hour. This might look painful but not as painful and seeing a patient with COVID-19 die alone, not as painful as holding an iPad for a patient and their loved ones so they can see each other, not as painful and talking to a patient's loved ones and telling them bad news, and not as painful as seeing people protesting. To the people on the frontline of this pandemic it is a slap in the face. **I have not been able to see the people I love for fear that I might inadvertently give them this disease.** Please stay at home, stay safe and take care of one another."
	April 2020, Algeria<br><br>"I'm scary ? Do I look like a zombie? Well, I am among the thousands of caregivers facing this health emergency.<br><br>I'm scared, not for me because as much as a caregiver, I'm basically vulnerable to many viruses on a daily basis, but **#covid19 is different, so I'm much more scared for my family, scared to go to work, scared that I accidentally touch my face... afraid of bringing this virus home, afraid of contaminating my family!**<br>I'm physically tired because the protective devices hurt, the suit makes me sweat and once dressed, I can't go to the toilet, drink or eat for 16 hours.<br>I am psychologically tired, like all my colleagues who have already been in the same state for weeks, say months, and were more exposed than me!<br>but that won't stop us from doing our job as we've always done, because yes we always respond to distress calls!<br><br>The requisitions have begun, without sufficient protective equipment (hamdoulilah kayn nas lkhir with their donations and fortunately I have already bought what is needed with my own means), with poor administrative management, non-solidarity staff because yes they do not all have a professional conscience, now I blame the people, the unconscious people who did not hear our alerts, our advice knowing that everyone knows the situation of our hospitals, you did not respect the barrier (sic social distancing) gestures , today I hold you all responsible, for all its victims, for all the infected, deceased caregivers... Today we are on the front line to flatten the curve of this virus and it still amazes me that people doubt the seriousness of this crisis (nas mazalha tokhrj for everything and anything...)

	I have done and I continue to do my part, do yours 🙏"
**Extreme emotional and physical consequences**	May 2020, Spain<br><br>"All of us health workers are very disappointed to see that as soon as the confinement measures have been eased, many people have done what they wanted. Flattening that curve with the lives it has laid down, with the sacrifice we are making to continue saving lives. If you saw what I've seen in the emergency room, you wouldn't play smart passing the guidelines where I know. Do you still remember that we risk our lives every day, we are exhausted, injured, with sleep problems, states of anxiety and stress? I think many people no longer even know why they go out to applaud at 8:00 p.m.<br>In my particular case, **I don't sleep at all, my hair falls out and it has turned gray, I lose weight, I have tachycardia when I go to sleep, I want to cry all the time, my hands and face hurt, I have hurt myself on my back from scratching due to stress and I have muscle contractures and pain all over my body, as well as an exhaustion that sometimes leaves me in bed unable to move and some problems that I will have to solve when this is over. And I'm not one of those who is worse....**<br>We all understand how difficult it is to stay home. I have a four year old who needs to go out. But let's do things right! Because if it goes up again or there is another regrowth, I don't know who will be there on the front line.<br>Now you can unfollow me, because every time I upload this stuff people seem to get offended. But I do not care. Let only those who truly value me remain."
	April 2020, USA<br><br>**"I've been trying to think of captions that truly explain how I feel in regards to everything going on right now. And every time I think about it it's just producing so much fucking anxiety, sadness, disappointment, fear...** this pandemic had such opportunity to empower nurses, collaborate, heal.. but instead **we are drowning... each day comes with less support & more responsibility. The safety & security we had yesterday is gone the next day. We have no legal support to back us up as we continue to lose our rights as a nurse, as a human. We can't escape the misery.**<br>**We go 13 hours at a time sweating in places we've never sweat before from the gowns & stale OR scrubs... faces & ears aching from the suffocating masks. Then we go & care for**

	**people who are dying alone, because there are no visitors allowed. The emotional burden truly rips at the caring heart of every nurse.**<br>And if we're lucky enough to get a break from it all, we're scared to eat lunch in such a contaminated workspace, for fear of dying amongst the lifeless bodies surrounding us. How ironic that would be. And that's all you think about. All shift.<br>When am I gonna get it.<br>My coworkers know my code status right?<br>Will I be the cause of my husbands death?<br>Kids?<br>**We haven't even hit our peak yet but we're told it's coming.** How envious we are of those "bored" people that are forced to have to work from home. But we continue to go to work because we have to. Deep down we want to.<br>We want to help. It's our calling. And I know that as nurses, nationally.. globally... we will never stop doing what we are called to do. And we will die trying."
	April 2020, Spain<br><br>"... **I don't care how many hours I spend in a box, with my glasses and mask on, with the great PPE's (irony) that they give us, being hot, overwhelmed, stressed because you get a ticket to the upac (one of the ICUs) and you know when you enter but never when you leave...** we work, we act as a team to give our best care to patients and to be able to save many lives from **this damn pandemic that is taking away our sleep, causing us anxiety, tachycardia, nerves, fear .** It is true that a warrior is not sent to war with a water pistol, they go with missiles and that is what we need adequate material and PPE's, so we cannot continue! **It affects us a lot. I just want this to end.**<br>And when it's over, all the health personnel will go out into the street to claim and demand that public health is not to be played with and I hope that you will also go out with us and continue to applaud us, cheering us on. Because that's when we'll need you the most!<br>**And this is the result from 2:45 a.m. to 7:54 a.m., even second-skin dressings do nothing for me."**
	April 2020, Italy

	“What is happening is something surreal, like a movie, yet this is the reality whether we like it or not ... This is the World we are living now, this is the difficulty we are facing now, not tomorrow, but now.<br>We are doing our best, all of us health workers are fighting every day on the front line against something invisible and difficult that is upsetting the daily life that we had before ... **Do not think that it is a walk for us to face a shift of I work in these conditions, physically and psychologically you come out almost destroyed, yet every day we are there, ready to fight again, for everyone, for Italy, for us and for you** ... We are not Heroes, We are human like you, but with Passion we have chosen a job that helps others especially in times of difficulty, for this reason we will never back down, and we will fight on the front line. But for this battle you can fight too, for this we ask you only for help, one simple thing:<br>STAY AT HOME ...<br>We ask it for YOU, for those you WANT WELL, for US and for our beautiful and beloved COUNTRY that is facing an enormous difficulty and that only with our sense of responsibility will it be possible to emerge victorious ...<br>Be Italian not selfish, be responsible not stupid.<br>STAY AT HOME !!!<br>WE'LL MAKE IT!!! 💪💪💪💪💪<br>IT WILL ALL BE FINE<br>ITITITIT [sic 4 italian flag emojis in a row]”
	March 2020, England<br><br>**“This is the face of someone who just spent 9 hours in personal protective equipment moving critically ill Covid19 patients around London.**<br><br>**I feel broken - and we are only at the start. I am begging people, please please do social distancing and self isolation.”**
**Creating a Collective Voice, the Shared COVID-19 Experience**	
	April 2002, England<br><br>**“Mine and my colleagues faces are sore and we are run ragged. Mostly we are desperately trying to save lives. Please please #StayHomeSaveLives”**
	March 2020, Italy

	"I am a nurse and I facing this health emergency right now. I'm scared too, but not to go grocery shopping, I'm afraid to go to work. I'm scared because the mask might not fit my face well, or I may have accidentally touched myself with dirty gloves, or maybe the lenses don't cover my eyes and something might have passed. I'm physically tired because protective devices hurt, scrubs are sweating and once dressed I can't go to the bathroom or drink for six hours. **I am psychologically tired, and as I am all my colleagues who have been in the same condition for weeks, but this won't stop us from doing our job like we have always done.** I will continue to care and take care of my patients, because I am proud and in love with my job. What I ask anyone reading this post is not to frustrate the effort we are making, to be selfless, to stay in the house and so protect those who are most fragile. We young people are not immune to coronavirus, we can get sick too, or worse we can make you sick. I can't afford the luxury of going back to my house in quarantine, I have to go to work and do my part. You all do [sic make] yours, I ask you please."
	<u>March 2020, UK</u><br><br>"... Today was my first shift helping out on Covid Intensive Care. Massive respect to the nurses who work in ICU all the time; it isn't easy (especially in full PPE)! To anyone who still isn't taking it seriously and thinks that the lockdown is over the top...it's not. You might not get as poorly as these people in ICU but someone you come into contact with could. Please don't be selfish. Enjoy spending more time with your family. Stay at home. Stay safe.<br>On a side note...**so proud of my NHS team for stepping up to face the challenge in these last few weeks. I am very lucky to work with some amazing people.** (Fingers crossed my face won't look like this post-covid 🙄) ."
	<u>April 2020, Spain</u><br><br>"... I don't care how many hours I spend in a box, with my glasses and mask on, with the great PPE's (irony) that they give us, being hot, overwhelmed, stressed because you get a ticket to the upac (one of the ICUs) and you know when you enter but never when you leave... we work, we act as a team to give our best care to patients and to be able to save many lives from this damn pandemic that is taking away our sleep, causing us anxiety, tachycardia, nerves, fear . It is true that a warrior is not sent to war with a water pistol, they go with missiles and that is what we need adequate material and PPE's, so we cannot continue! It affects us a lot. I just want this to end.

	And **when it's over, all the health personnel will go out into the street to claim and demand that public health is not to be played with and I hope that you will also go out with us and continue to applaud us, cheering us on. Because that's when we'll need you the most!**<br>And this is the result from 2:45 a.m. to 7:54 a.m., even second-skin dressings do nothing for me.”
	April 2020, USA<br><br>“I’ve been trying to think of captions that truly explain how I feel in regards to everything going on right now. And every time I think about it it’s just producing so much fucking anxiety, sadness, disappointment, fear... this pandemic had such opportunity to empower nurses, collaborate, heal.. but instead we are drowning... each day comes with less support & more responsibility. The safety & security we had yesterday is gone the next day. We have no legal support to back us up as we continue to lose our rights as a nurse, as a human. We can’t escape the misery.<br>We go 13 hours at a time sweating in places we’ve never sweat before from the gowns & stale OR scrubs... faces & ears aching from the suffocating masks. Then we go & care for people who are dying alone, because there are no visitors allowed. The emotional burden truly rips at the caring heart of every nurse.<br>And if we’re lucky enough to get a break from it all, we’re scared to eat lunch in such a contaminated workspace, for fear of dying amongst the lifeless bodies surrounding us. How ironic that would be. And that’s all you think about. All shift.<br>When am I gonna get it.<br>My coworkers know my code status right?<br>Will I be the cause of my husbands death?<br>Kids?<br>We haven’t even hit our peak yet but we’re told it’s coming. How envious we are of those “bored” people that are forced to have to work from home. But **we continue to go to work because we have to. Deep down we want to.**<br>**We want to help. It’s our calling. And I know that as nurses, nationally.. globally... we will never stop doing what we are called to do. And we will die trying.”**
